# Numerical Modeling of Silicon Photodiodes for High-Accuracy Applications Part III: Interpolating and Extrapolating Internal Quantum-Efficiency Calibrations

**DOI:** 10.6028/jres.096.025

**Published:** 1991

**Authors:** Jon Geist, A. M. Robinson, C. R. James

**Affiliations:** National Institute of Standards and Technology, Gaithersburg, MD 20899; University of Alberta, Alberta, Canada T6G 2G7

**Keywords:** extrapolation, internal quantum-efficiency, interpolation, photodiode, silicon, visible

## Abstract

The semiconductor device modeling program PC-ID and the programs that support its use in high-accuracy modeling of photodiodes, all of which were described in Part I of this series of papers, are used to simulate the interpolation of high-accuracy internal quantum-efficiency calibrations in the spectral region between 450 nm and 850 nm. Convenient interpolation formulae that depend only upon wavelength are derived. Uncertainty spectra for a number of sources of error are also derived. The formulae are normalized to experimental internal-quantum efficiency calibrations in the 440 to 470 nm spectral region and at 860 nm and arc used to interpolate the calibration values between these wavelengths. The results of the interpolations are compared with experimental calibration data that are available at a few wavelengths between 440 and 860 nm. The disagreement between the interpolated and measured internal quantum-efficiency data is never worse than 0.0003.

## 1. Introduction

Part II of this series of papers suggested that the Hamamatsu^1^ 1337 type photodiode might be conveniently used for interpolating or extrapolating high-accuracy quantum efficiencies in the 400 to 900 nm spectral region. The idea of quantum-efficiency extrapolation was implicit in the first detailed description of how a silicon photodiode could be used (in this case in conjunction with a thermal detector having a spectrally flat responsivity) to realize a scale of absolute spectral responsivity [1], and interpolation was explicitly considered in reference [2], Beyond these two publications, however, little progress has been reported in this area until recently.

Hoyt et al. [3] (using early results of the work described here) used three Hamamatsu 1337 type photodiodes to extrapolate a quantum-efficiency calibration obtained at 441.6 nm with a UDT QED 100 radiometer to 633.0 nm, and reported an average difference of 0.04% compared to independent measurements based on their electrically calibrated cryogenic cavity radiometer. Even though 0.04% is quite good by conventional radiometric standards, it was a factor of two larger than the combined uncertainty estimate for the two measurement techniques being compared, and further study was proposed by Hoyt et al. More recently, Zalewski and Hoyt [4] have reported a more direct test of the accuracy of the use of the Hamamatsu 1337 type photodiode to extrapolate quantum-efficiency values. The results of this test agreed to within the combined estimated uncertainty, and two errors were discovered in the earlier work. When these errors were corrected, agreement within the estimated uncertainty of the earlier intercomparison was obtained.

The purpose of Part III of this series of papers is to develop formulae and uncertainty estimates for interpolating and extrapolating internal quantum-efficiency calibrations with Hamamatsu 1337 photodiodes. It will be shown that this type of photodiode has characteristics that suit it particularly well for this task over the 440 to 860 nm spectral region. The remainder of Part III is organized as follows: Section 2 investigates the errors associated with the use of relations that are only approximate for the 1337 type photodiode, but which simplify interpolation and extrapolation of the internal quantum deficiency (one minus the internal quantum efficiency) for this type of photodiode. Section 3 investigates the various sources of error associated with the actual calculation of internal quantum-deficiency spectra using the semiconductor-device modeling program PC-1D and the support programs described in Part I. Finally, section 4 compares the results obtained here with experimental measurements and demonstrates agreement within 0.0003.

## 2. Errors Associated with the Quantum-Deficiency Approximations

For the purposes of extrapolating and interpolating the internal quantum efficiency of Hamamatsu 1337 type photodiodes, the internal quantum deficiency can be approximated by a function δ(λ,*N S*,*τ*_r_) of wavelength λ and of three recombination-related variables: *N_ss_* the charge number density stored in the front-surface oxide, *S* the surface recombination velocity at the oxide-silicon interface, and *τ*_r_ the minority-carrier lifetime in the rear region.

For silicon photodiodes, δ(λ,*N*_ss_,*S*,*τ*_r_) can be approximated by a function that has properties that make it particularly useful for extrapolating and interpolating quantum-deficiency measurements. The approximate equation for δ(λ,*N*_ss_,*S*,*τ*_r_) is

$$\begin{array}{l} \delta \left(\lambda ,N_{\mathrm{ss}},S,\tau_{r}\right)=K_{f}\left(N_{\mathrm{ss}},S\right)\delta_{f}\left(\lambda \right)\\ +K_{r}\left(\tau_{r}\right)\delta_{r}\left(\lambda \right), \end{array}$$ (1)

where

$$\delta_{r}\left(\lambda \right)=0,$$ (2)

for λ less than some wavelength λ_0_.

To see how this is useful for interpolating quantum-deficiency values, suppose that δ(λ_f_,*N*_ss_,*S*,*τ*_r_) has been measured at wavelength λ_f_< λ_0_, and δ(λ_f_, *N*_ss_,*s,τ*_r_) has been measured at wavelength λ_f_>λ_0_. The values for *K*_f_(*N*_ss_,*S*) and *K*_r_(*τ*_r_) can then be determined from

$$K_{f}\left(N_{\mathrm{ss}},S\right)=\frac{\delta \left(\lambda_{f},N_{\mathrm{ss}},S,\tau_{r}\right)}{\delta_{f}\left(\lambda_{f}\right)},$$ (3a)

and

$$K_{r}\left(\tau_{r}\right)=\frac{\delta \left(\lambda_{r},N_{\mathrm{ss}},S,\tau_{r}\right)-K_{f}\left(N_{\mathrm{ss}},S\right)\delta_{f}\left(\lambda_{r}\right)}{\delta r\left(\lambda_{r}\right)},$$ (3b)

and these values can be used in eq (1) to interpolate the quantum deficiency between λ_f_ and λ_r_. In the above equations, δ_f_(λ) and δ_r_(λ) are functions only of wavelength, λ_f_ and λ_r_ are the wavelengths at which the internal quantum deficiency is to be normalized, and λ_0_ will generally fall between 600 and 650 nm. The subscript f stands for the front of the photodiode since this is the location of the recombination mechanisms that cause δ(λ) to exceed zero. The subscript r stands for the rear of the photodiode since this is the location of the recombination mechanism that cause δ_r_(λ) to exceed zero. Therefore, δ_f_(λ) and δ_r_(λ) will be called the front-region and rear-region internal quantum-deficiency spectra, respectively. The functional dependence of *K*_f_ and *K*_r_ will be dropped from the notation for the remainder of this paper.

**Figure 1:**
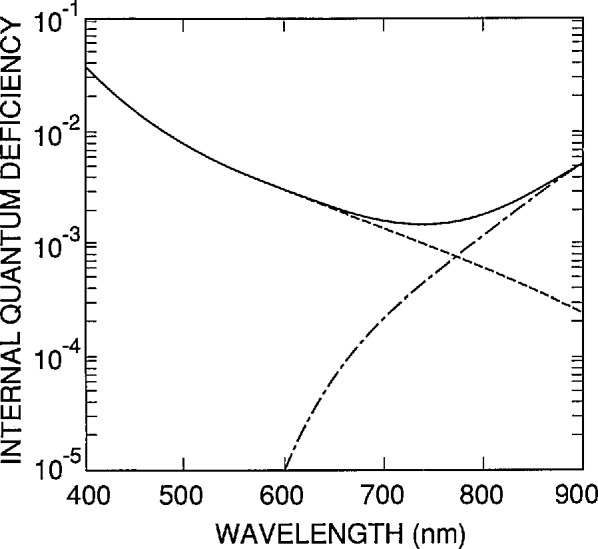
The quantum-deficiency spectra δ(λ,0,71264 cm/s, ∞) (dashed line) and δ(λ,0,0,1 ms) (dot-dashed line) and their sum (solid line).

Exactly how accurate eqs (1–3) are depends upon the particular type of photodiode and the range covered by the parameters. Figure 1 illustrates the accuracy of eq (1) for the 1337 type photodiode (modeled as described in Part II of this series of papers) over the 400 to 900 nm spectral region for an internal quantum-deficiency spectrum that is a practical upper limit for this type of photodiode. Figure 1 compares the internal quantum-deficiency spectra δ(λ,0,71264 cm/s, ∞) and δ(λ,0,0,1 ms) with their sum, and with the internal quantum-deficiency spectrum δ(λ,0,71264 cm/s, 1 ms). The latter spectrum and the sum of the two first spectra differ by less than 2×l0^−5^ over the entire 400 nm spectral region, and cannot be distinguished at the scale of the figure. (Any errors with absolute values less than 2 × 10^−5^ are negligible for the purposes of this paper.)

**Figure 2:**
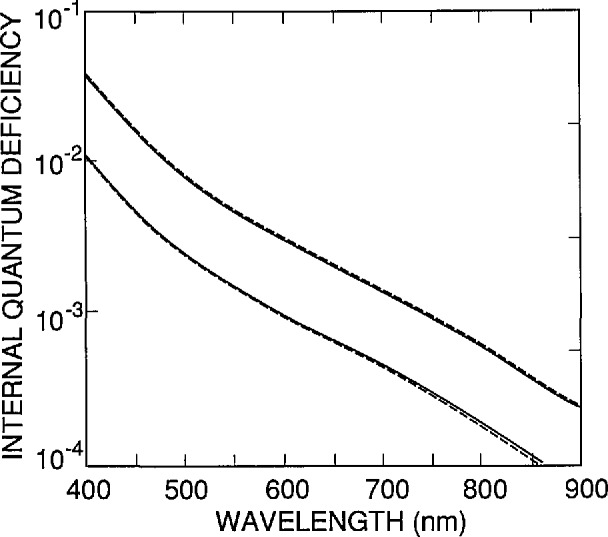
The quantum-deficiency spectra δ(λ,*N*_ss_,*S*,τ_r_) for the conditions (Case 1 –upper dashed line; Case 2 –lower dashed line; Case 3 –upper solid line; Case 4 –lower solid line) listed in table 1.

**Figure 3:**
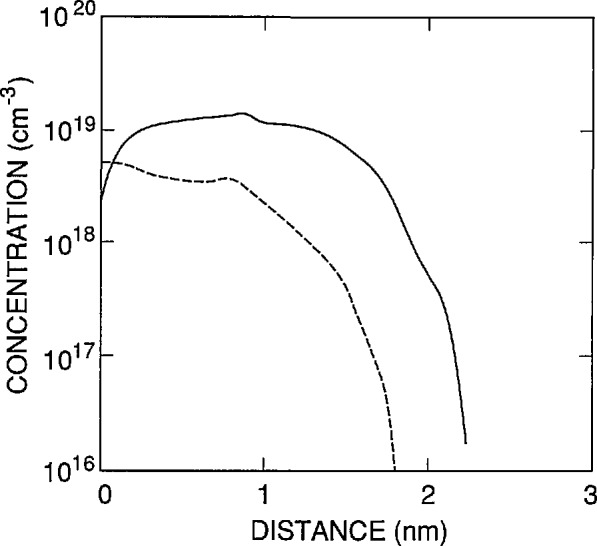
The front-region doping distributions *N*_A_(*x*) used to calculate the front-region internal quantum-deficiency curves in figure 2.

Figure 2 illustrates the accuracy of eqs (1–3) over the 400 to 900 nm spectral region for the case where τ_r_ = ∞. The spectra in figure 2 were calculated for the range of parameters listed in table 1. Cases 1–3 in that table are based on the front-region doping distribution *N*_A_(*x*)/*N*_A_(0) shown as the dashed curve in figure 3, and Case 4 is based on that shown as the solid curve in the same figure. Of six doping distributions measured for different 1337 type photodiodes, the two shown in figure 3 produce the largest change in shape of the internal quantum-deficiency spectrum.

**Figure 4:**
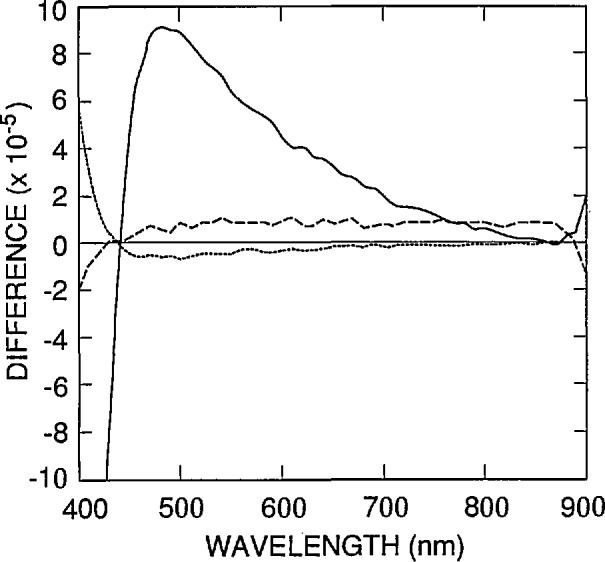
Differences between the internal quantum-deficiency spectra of figure 2 for Cases 1 (dashed line), 3 (dotted line), and 4 (solid line) when normalized to 0.01 at 440 nm, and that for Case 2 when normalized to 0.01 at 440 nm.

The shapes of the front-region internal quantum-deficiency spectra in figure 2 are compared in figure 4 by normalizing all of the spectra to 0.01 at 440 nm and by plotting their differences relative to the normalized spectrum for Case 2. Cases 1 and 3 compared with Case 2 shows how the shape of δ_f_(λ) changes when its magnitude is increased by over a factor of three in association with either a substantial decrease or a substantial increase in *N*_ss_. (Cases 1 and 3 bracket a number of cases that were examined in which *N*_ss_ and *S* were independently varied within the range of values shown in table 1.)

Case 4 compared with Case 2 shows how the shape of δ_f_(λ) changes when the dopant distribution is changed from the dashed curve to the solid curve in figure 4. This effect is much larger than that associated with varying *N*_ss_ and *S.*

The three spectra in figure 4 characterize the uncertainties to be associated with the diode-to-diode variations in *N*_ss_, *S*, and *N*_ss_(*x*) when any one of the spectra in that figure is used as δ_f_(λ) with *K*_f_=0.01 in eqs (1) and (3). These spectra should be multiplied by *K*_f_/0.01 if *K*_f_≠0.01. For the purposes of this paper, the δ(λ,*N*_ss_,*S*,∞) spectrum corresponding to Case 4 in table 1 will be used for δ_f_(λ) in eqs (1) and (3).

The front-region internal quantum deficiency also depends upon two front-region recombination mechanisms not considered above. These are the Auger and band-to-band mechanisms that become important in the heavily doped front region of the photodiode. Both effects were included in all of the spectra described so far by using the default cross sections that are built into PC-1D for these recombination mechanisms. The PC-1D default values for the Auger recombination cross sections give an internal quantum-deficiency spectrum with a value of 9×10^−6^ at 400 nm. The shape of this spectrum is well modeled by δ_f_(λ) [5]. In fact, an uncertainty of ±100% can be tolerated in the Auger cross sections without causing an error as large as 2 times 10^−5^ anywhere within the 400 to 900 nm spectral region.

**Figure 5:**
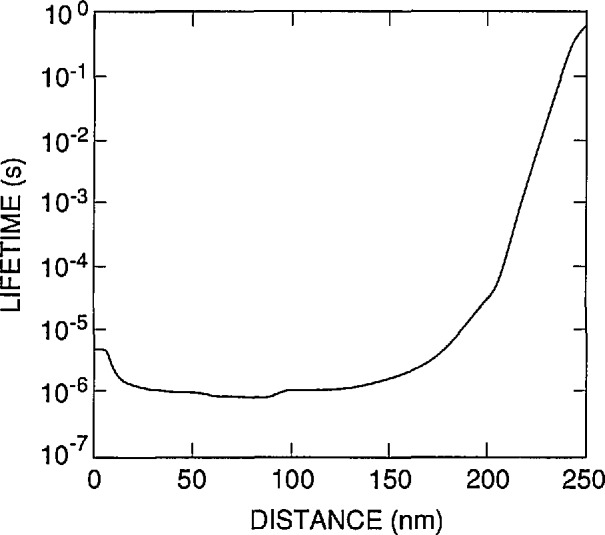
The default, band-to-band recombination lifetime profile calculated by PC-1D for the doping distribution shown as the solid curve in figure 3.

For the doping profile shown as the solid curve in figure 3, the PC-ID default values for the band-to-band recombination cross sections give the lifetimes shown in figure 5 as a function of distance from the oxide-silicon interface in the photodiode. These lifetimes give an internal quantum-deficiency spectrum with a value of 6×10^−5^ at 400 nm. This spectrum is not as well modeled by δ_f_(λ) as is the spectrum for Auger recombination, but an uncertainty of ±50% in the band-to-band recombination cross sections can be tolerated without raising the error associated with this uncertainty to 2×l0^−5^ anywhere in the 400 to 900 nm spectral region.

**Figure 6:**
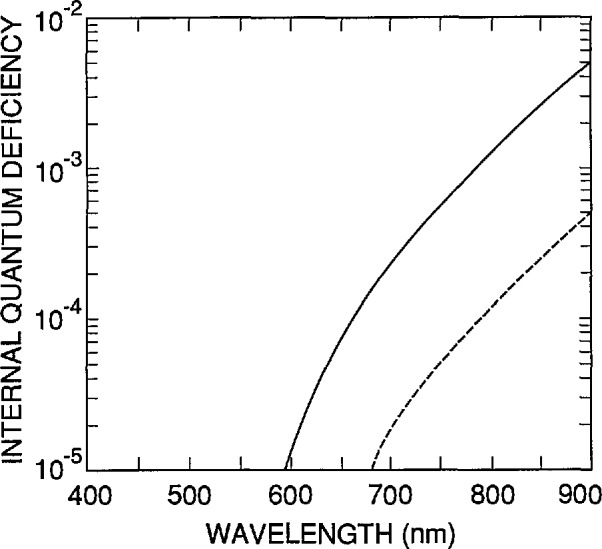
The internal quantum-deficiency spectra δ(λ,*N*_ss_,*S*,τ_r_) for t_r_= 1 ms (solid line), and τ_r_=10 ms (dashed line) with *N*_ss_=*S*=0.

**Figure 7:**
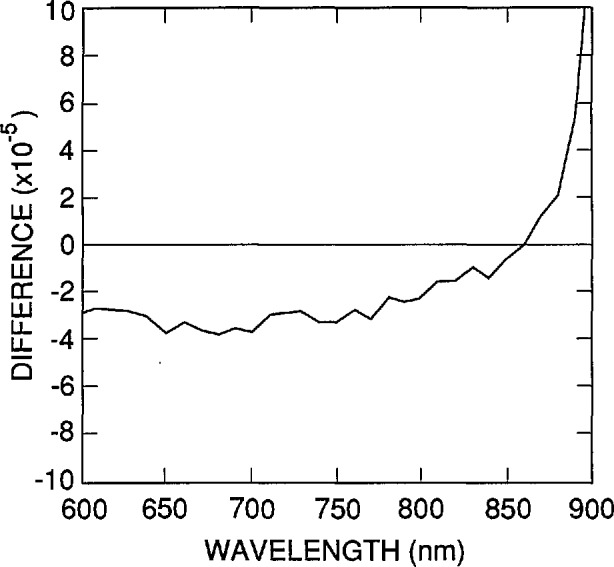
Differences between the internal quantum-deficiency spectra of figure 6 when normalized to 0.002 at 860 nm.

Now consider δ_r_(λ). Figures 6 and 7 illustrate the accuracy of eqs (1) and (3) over the 400 to 900 nm spectral region for the case where *S*=0. (Note that when *S*=0, the internal quantum-deficiency spectrum is independent of *N*_ss_.) The spectra in figure 6 were calculated for τ_r_=1 ms and 10 ms, with *N*_ss_=*S*=0. For the purposes of this paper, δ_r_(λ) will be set to zero in eqs (2) and (3) for λ≤610 nm, and the spectrum for τ_r_= 1 ms will be used for δ_r_(λ) for λ > 610 nm.

The shapes of the spectra in figure 6 are compared in figure 7 by normalizing them to 0.002 at 860 nm and plotting their differences relative to the spectrum for τ_r_= 1 ms. The normalization value of 0.002 was chosen as a practical upper limit. The difference spectrum in figure 7 characterizes the uncertainties to be associated with the variations in rear-region lifetime when the spectrum for τ_r_ = l ms is used as δ_r_(λ) with *K*_r_= 0.002 in eqs (2) and (3). This spectrum should be multiplied by *K*_r_/0.002 if *K*_r_≠0.002.

A convenient function of wavelength has been fitted to the spectrum corresponding to Case 4 in table 1, and another to the spectrum for τ_r_ = 1 ms in figure 6. These functions are

$$\delta_{f}\left(\lambda \right)=A_{0}\left[X\left(\lambda \right)+A_{1}X^{2}\left(\lambda \right)+A_{2}Y\left(\lambda \right)\right],$$ (4)

and

$$\delta_{r}\left(\lambda \right)=A_{0}\left[1/X\left(\lambda \right)-A_{2}/Y\left(\lambda \right)\right],$$ (5)

respectively, where

$$X\left(\lambda \right)=\exp\left(-\lambda /\lambda_{0}\right),$$ (6)

and

$$Y\left(\lambda \right)=\exp\left(-{\left(\lambda /\lambda_{2}\right)}^{2}\right).$$ (7)

The appropriate values for *A*_0_, *A*_1_ *A*_2_, λ_0_, and λ_2_ for eqs (4) and (5) are listed in table 2. The function in eq (5) becomes negative for λ < 620 nm, in which case eq (5) is replaced with δ_r_(λ) = 0 as mentioned earlier. The values of *A*_0_ in table 2 normalize δ_f_(λ) to 0.01 at 440 nm, and δ_r_(λ) to 0.002 at 860 nm, respectively.

With *K*_f_=0.5516405, eq (4) fits the spectrum for *N*_ss_= −3×10^12^ cm/s in figure 4 with a residual standard deviation of 2.9×10^−6^ over the 420 to 900 nm spectral region and never differs from that spectrum by more than 7×10^−6^ over the same spectral region. This translates to less than 10 ppm when the internal quantum deficiency is normalized to 0.01 at 440 nm; thus the error associated with the use of eq (4) is negligible for the purposes of this paper. The larger variations below 420 nm are negligible with respect to uncertainties already identified and others discussed below.

With *K*_r_= 1.685081, eq (5) fits the spectrum shown as a solid line in figure 6 with a residual standard deviation of 1.4×10^−5^ over the 610 to 900 nm spectral region, and never differs from that spectrum by more than 2.5×10^−5^ over that same spectral range. This translates to less than 1.7×10^−5^ when the quantum deficiency is normalized to 0.002 at 440 nm; thus the error associated with the use of eq (5) is also negligible for the purposes of this paper.

## 3. Errors Associated with Quantum-Deficiency Calculations

Two types of error associated with the quantum-deficiency values calculated by PC-1D can be distinguished: 1) numerical errors associated with the algorithms used by PC-1D and the limited number of finite elements available to PC-1D, and 2) physical errors associated with approximations and simplifications in the physical models used with, or built into, PC-1D. Both types are considered in this section.

It was shown in reference [5] that δ_f_(λ) is well approximated by

$$\delta_{f}\left(\lambda \right)=\frac{\Gamma \left\{I\left(0\right)-I\left[\alpha \left(\lambda \right)\right]\right\}}{1+\Gamma I\left[\alpha \left(\lambda \right)\right]},$$ (8)

$$I\left[\alpha \left(\lambda \right)\right]\int_{0}^{x_{1}}\hat{k}\left(x\right)\exp\left(-\alpha \left(\lambda \right)x\right)dx,$$ (9)

$$\hat{k}\left(x\right)=k\left(x\right)/k\left(0\right),$$ (10)

$$k\left(x\right)=1/\left[m_{0}\left(x\right)D_{m}\left(x\right)\right],$$ (11)

$$\Gamma =S/D_{m}\left(0\right),$$ (12)

where α(λ) is the absorption-coefficient spectrum of silicon, *x* is the distance from the oxide-silicon interface toward the rear of the photodiode, *m*_0_(*x*) is the equilibrium minority-carrier concentration at the point *x* in the front region of the photodiode, and *D_m_*(*x*) is the equilibrium diffusion constant for the minority carriers at the point *x.* Note that the sign in the numerator in eq (8) is correct and that the sign is incorrect in reference [5]. Also, note that *f*(λ) becomes very insensitive to *x*_1_ as *x*_1_ increases beyond some critical value. The point where the electron and hole concentrations cross is a convenient choice for *x*_1_, but smaller values would work as well.

From the point of view of PC-1D, the kernel k(*x*) in eq (11) is given by

$$k\left(x\right)=qM_{0}\left(x\right)/\left[n_{ie}^{2}\left(x\right)kT\mu_{m}\left(x\right)\right],$$ (13)

where *M*_0_(*x*) is the equilibrium majority carrier concentration in the front region, *n*_ie_(*x*) is the effective intrinsic carrier concentration [6], which in PC-1D includes the effect of Fermi-Dirac statistics, *µ*_m_(*x*) is the minority-carrier mobility as a function of position in the front region, *k* is the Boltzmann constant, and *T* is the temperature of the photodiode.

**Figure 8:**
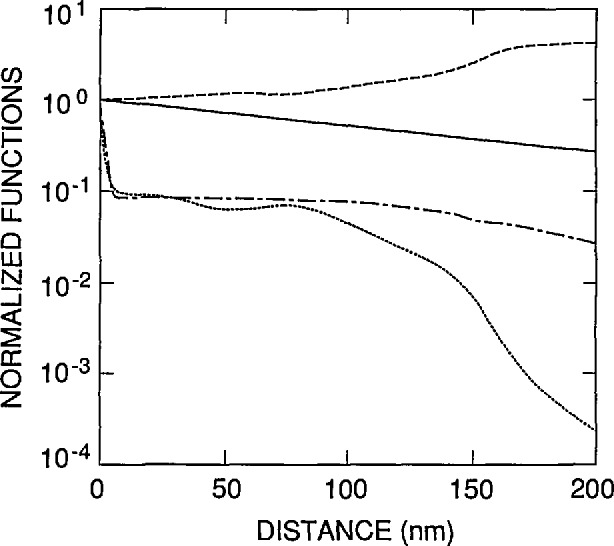
Comparison of ${\hat{M}}_{0}\left(x\right)$ (dotted line), ${\hat{n}}_{\mathrm{ie}}\left(x\right)$ (dashed line), $\hat{\mu }$ (dot-dashed line), and exp(−α(400 nm) *x*) (solid line) for a 1337 type photodiode with *N*_ss_, *S*, and τ_r_, set as defined in Case 2 of table 1. The tildes in the preceding expressions indicate that these expressions have been normalized to unity at *x* =0, as shown for *k*(*x*) in eq (10).

Figure 8 compares exp(−α(400 nm)*x*) with the equilibrium functions *M*_0_(*x*), *n*_ie_(*x*), and *µ*_m_(*x*) as calculated by PC-1D over the range 0< *x* <200 nm for the same photodiode model used to calculate the quantum-deficiency spectra in figure 3 for Case 2 of table 1. Equations (8–12), figure 8, and figure 3 show why the shape of δ_f_(λ) is very insensitive to *N*_ss_ while being quite sensitive to the shape of *N*_A_(*x*)/*N*_A_(0). Since the width of the accumulation layer created by *N*_ss_ is small compared to the distance over which exp(−α(λ)*x*) changes significantly for λ ≥400 nm, changes in *N*_ss_ have the effect of multiplying the integral in eq (9) by a constant factor independent of λ≥400 nm. On the other hand, the changes in *N*_A_(*x*)/*N*_A_(0) shown in figure 3 extend well into the region where exp(−α(λ)*x*) varies significantly from unity for λ = 400 nm. As λ is increased above 400 nm, the shape of δ_f_(λ) will become less dependent on the shape of *N*_A_(*x*)/*N*_A_(0) because α(λ) decreases with increasing λ.

The numerical accuracy with which PC-1D calculates *M*_0_(*x*) within the framework of the drift-diffusion approximations for the conditions of charge accumulation at an oxide-silicon interface as shown in Figure 8 has been studied previously and found to be quite good [7]. With a uniform doping density of 10^19^ cm^−3^ and an oxide charge density of 10^13^ cm^−2^, PC-1D overestimates *M*_0_(*x*) by about 9% at *x* = 0, decreasing to within ±1% for *x* > 1 nm. The uncertainty in *k*(*x*) due to this source of error will be modeled by replacing *k*(*x*) by

$$k_{1}\left(x\right)=k\left(x\right)/\left(1-0.1x/x_{0}\right),$$ (14)

for *x <x*_0_, and

$$k_{1}\left(x\right)=k\left(x\right)/0.9,$$ (15)

for *x* >*x*_0_ in the kernel of the integral in eq (9), where *x*_0_ = 1 nm.

Another source of error that must be considered is the numerical accuracy with which PC-1D would calculate the steady-state photocurrent if the equilibrium carrier concentration were exact. The uncertainty associated with this source of error was estimated as follows: 1) The internal quantum-deficiency spectrum was calculated directly from the PC-1D solution of the steady-state drift-diffusion equations for the total current flowing in the photodiode for the conditions described by Case 2 of table 1. 2) Equations (8–13) were used to calculate the internal quantum-deficiency spectrum from the equilibrium values of *M*_0_(*x*), *n*_ie_(*x*), and *µ*_m_(*x*) calculated by PC-1D for the same conditions as in 1) above. The integration in eq (9) was carried out using a generalization of Simpson’s rule for nonequidistant points. 3) Both spectra were normalized to 0.01 at 440 nm, and the differences calculated. There is no reason to believe that these differences, which exceed 2×10^−5^ only at 400 and at 900 nm, are caused by errors associated with PC-1D. It is just as likely that they are associated with eqs (8–12) since these equations were derived from a number of assumptions that are not rigorously satisfied. The important point is that it is unlikely that either calculation is in error by much more than the differences between them. This verifies the numerical accuracy of the photocurrents calculated by PC-1D to the level of accuracy required in this paper.

There are a number of other errors, which are associated with incomplete or approximate physical models built into PC-1D, that also affect the accuracy with which PC-1D calculates the functions defining *k*(*x*) in eq (13). These include quantum-mechanical effects (tunneling) [8] that change the shape of *M*_0_(*x*) from that calculated from the drift-diffusion approximation for *x* <1 nm, band-gap narrowing effects [9] associated with heavy doping and large surface-fields [10] that cause *n*_ie_(*x*) [6] to depend upon position in the photodiode, and carrier-carrier and carrier-ion scattering mechanisms that cause *µ*_m_(*x*) to depend upon position [11]. It is beyond the scope of this paper to investigate each of these sources of error in detail, but the uncertainty in *k*(*x*) due to this source of error will be modeled by replacing *k*(*x*) by

$$k_{2}\left(x\right)=k\left(x\right)\left\{1-0.1\;\log\left[\hat{k}\left(x\right)\right]\right\}$$ (16)

in the kernel of the integral in eq (9). This allows an error of 10% per decade change in *k*(*x*).

The differences between the internal quantum-efficiency spectrum calculated from eqs (8–13) by replacing *k*(*x*) with either *k*_1_(*x*) or *k*_2_(*x*) in eq (9) and the spectrum calculated using *k*(*x*) were calculated after all three spectra were normalized to 0.01 at 440 nm. Neither difference exceeded 2×10^−5^ over the entire 400 to 900 nm spectral region. Therefore, all of these sources of error are considered negligible for the purposes of this paper.

**Figure 9:**
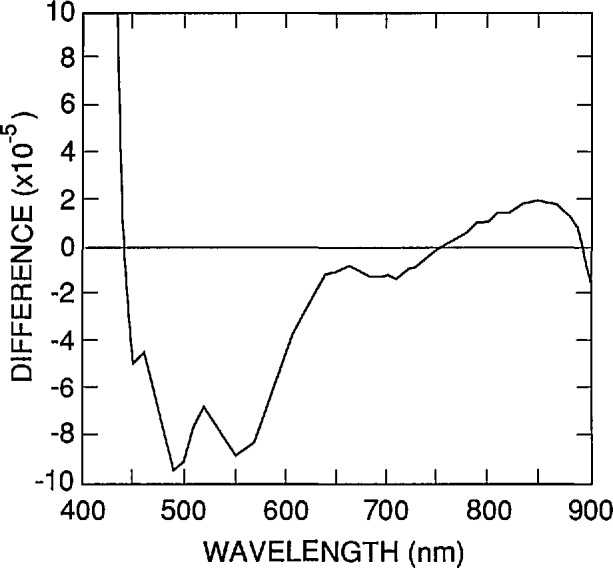
Differences between the internal quantum-deficiency spectrum for a 1337 type photodiode satisfying the conditions defined in table 1 for the absorption-coefficient data of reference [14] and for the absorption-coefficient data of reference [12].

**Figure 10:**
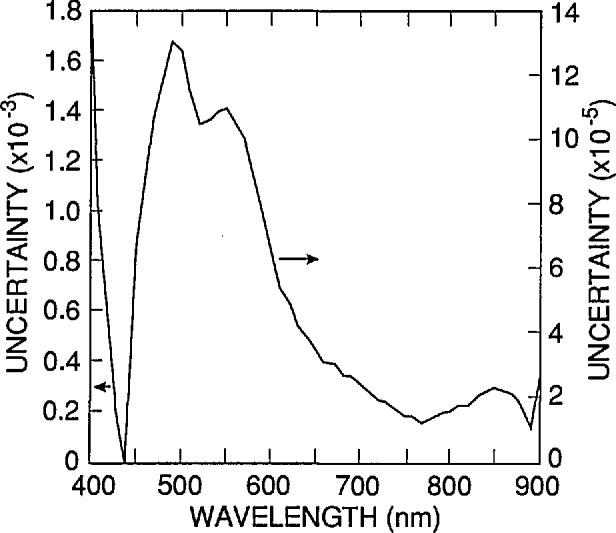
Differences between the internal quantum-deficiency spectra for a 1337 type photodiode with *S*=0 and *τ*_r_=1 ms for the absorption-coefficient data of reference [14] and for the absorption-coefficient data of reference [12].

Another source of error that is obvious in eqs (8–12) is the uncertainty associated with the absorption-coefficient data used in the simulations. As described in Part I, the absorption-coefficient data were calculated from an equation [12] fitted to the data of Weakliem and Redfield [13]. An alternate set of absorption-coefficient data was described by Philipp [14]. Figure 9 plots the differences between the internal quantum-deficiency spectra normalized to 0.01 at 440 nm for Case 2 of table 1 when based on the absorption-coefficient data in reference [14] and when based on the equation in reference [12]. These differences will be used as the estimated uncertainties arising from the uncertainties in the silicon absorption-coefficient spectrum when δ_f_(λ) is used to extrapolate an internal quantum deficiency of 0.01 from 440 nm to longer wavelengths with a 1337 type photodiode. A similar calculation was carried out for the case where *S*=0 and τ_r_ = 1 ms. Both spectra were normalized to 0.002 at 860 nm. The differences, which are shown in figure 10, are taken as the estimated uncertainties arising from the uncertainties in the silicon absorption-coefficient spectrum where δ_r_(λ) is used to extrapolate an internal quantum deficiency of 0.002 from 860 nm to shorter wavelengths with a 1337 type photodiode.

**Figure 11:**
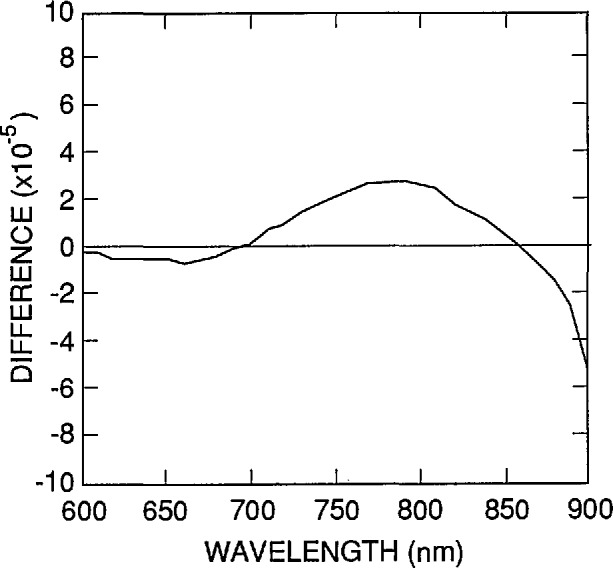
Sum in quadrature of the curves in figures 4 and 9. This curve is the nominal uncertainty associated with the use of eq (4) in eq (2) to extrapolate a quantum deficiency of 0.01 at 440 nm to any other wavelength between 400 and 900 nm. Notice that the sum is plotted on different scales below and above 440 nm.

**Figure 12:**
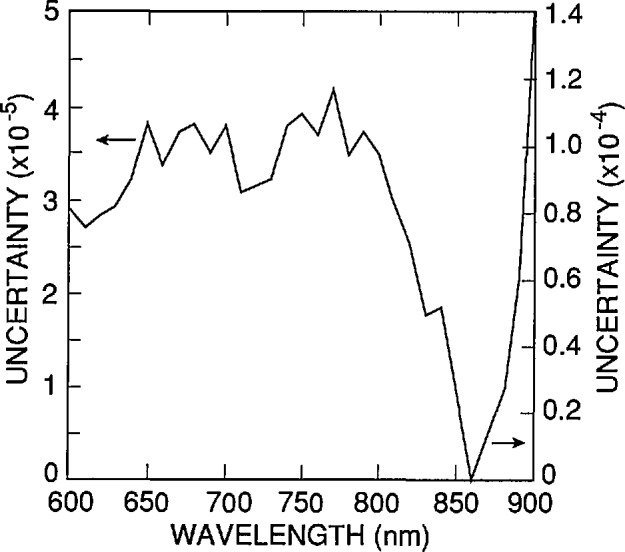
Sum in quadrature of the curves in figures 7 and 10. This curve is the nominal uncertainty associated with the use of eq (5) in eq (2) to extrapolate a quantum deficiency of 0.02 at 860 nm to any other wavelength between 400 and 900 nm. Notice that the sum is plotted on different scales below and above 440 nm.

Figures 11 and 12 plot the quadrature sum of the difference spectra plotted in figures 4 and 9 and in figures 7 and 10, respectively. The spectrum in figure 11 plots the estimated (one standard deviation) uncertainty as a function of wavelength that is associated with the use of δ_f_(λ) in eq (4) to extrapolate a measured internal quantum deficiency of 0.01 from 440 nm to longer wavelengths. The major sources of error contributing to the uncertainty spectrum in figure 11 are the diode-to-diode variations in *N*_A_(*x*)/*N*_A_(0) and the uncertainty in the absorption-coefficient spectrum for silicon, but a number of other sources of error were identified with the help of eqs (8–12), and any that produced differences greater than 2×l0^−5^ for 400 nm < λ <900 nm are included in figure 11.

The spectrum in figure 12 plots the estimated (one standard deviation) uncertainty as a function of wavelength that is associated with the use of δ_r_(λ) in eq (5) to extrapolate a measured internal quantum deficiency of 0.002 from 860 nm to shorter wavelengths. The major sources of error contributing to the uncertainty spectrum in figure 12 are the change in shape of δ_r_(λ) with τ_r_ and the uncertainty in the absorption-coefficient spectrum for silicon. All other sources of error considered were negligible with respect to 2×10^−5^ for 400 nm < λ <900 nm.

No equivalents to eqs (8−12) were used to guide the error analysis for δ_r_(λ). The results of the uncertainty analysis of δ_f_(λ) show that numerical accuracy of PC-1D will be a negligible source of error. This leaves the errors associated with the approximations in the physical models that are built into PC-1D. Some of these were tested, but no tests were possible for the use of a single SRH trap level to model the recombination in the rear region of the photodiode. This approximation could be a non-negligible source of error; it was shown in Part I that this approximation introduced errors of the order of the effect being modeled in nonlinearity simulations on a UV444B type photodiode. A more complete model might make τ_r_ depend upon position in the photodiode, which might modify the shape of δ_r_(λ) and add an uncertainty that is not included in figure 12.

## 4. Comparison with Experiment

To use the results developed thus far, it is necessary to define the internal quantum-deficiency interpolating function δ(λ), and to assume that it exactly satisfies eqs (1–3), which can be rewritten in more compact form as

$$\begin{array}{l} \delta \left(\lambda \right)=\\ \delta_{x}\left(\lambda_{f}\right)\frac{\delta_{f}\left(\lambda \right)}{\delta_{f}\left(\lambda_{f}\right)}+\delta_{x}\left(\lambda_{r}\right)\left[1-\frac{\delta_{f}\left(\lambda_{r}\right)}{\delta_{r}\left(\lambda_{r}\right)}\right]\frac{\delta_{r}\left(\lambda \right)}{\delta_{r}\left(\lambda_{r}\right)}, \end{array}$$ (17)

where δ*_x_*(λ_f_) and δ*_x_*(λ_r_) are the measured values of the internal quantum deficiency at the wavelengths λ_f_ and λ_r_, respectively; δ_f_(λ) and δ_r_(λ) are defined in eqs (4) and (5), respectively, and the constants used in these equations are defined in table 2. The uncertainty associated with δ(λ) due to the sources of error considered in the last two sections of this paper can be obtained by summing in quadrature each of the terms on the right-hand side of the differential of eq (17). That differential is given by

$$\begin{array}{l} d\delta \left(\lambda \right)=d\delta_{x}\left(\lambda_{f}\right)\left[\frac{\delta_{f}\left(\lambda \right)}{\delta_{f}\left(\lambda_{f}\right)}\right]\\ +d\delta_{x}\left(\lambda_{r}\right)\left[\frac{\delta_{r}\left(\lambda \right)}{\delta_{r}\left(\lambda_{r}\right)}\right]\;\left[1-\frac{\delta_{f}\left(\lambda_{r}\right)}{\delta_{r}\left(\lambda_{r}\right)}\right]\\ +d\delta_{f}\left(\lambda \right)\left[\frac{\delta_{x}\left(\lambda_{f}\right)}{\delta_{f}\left(\lambda_{f}\right)}\right]\\ +d\delta_{r}\left(\lambda \right)\left[\frac{\delta_{x}\left(\lambda_{r}\right)}{\delta_{r}\left(\lambda_{r}\right)}\right]\;\left[1-\frac{\delta_{f}\left(\lambda_{r}\right)}{\delta_{r}\left(\lambda_{r}\right)}\right]. \end{array}$$ (18)

The differentials *d*δ_f_(λ) and *d*δ_r_(λ) are plotted in figures 11 and 12 for λ_f_=440 nm, and for λ_r_ = 860 nm, respectively.

There are not many data available against which to test eqs (17) and (18), but there are some. For instance, Zalewski and Hoyt [4] have reported the internal quantum efficiencies at 441.6 nm and the spectral responsivities and absorptances at 633.0 nm for five multiple reflection (trap) [4,15–17] radiometers based on Hamamatsu 1337 photodiodes at 441.6 and 633.0 nm. Similarly, Fox [17] has reported the average internal quantum efficiency of 10 trap radiometers also based on Hamamatsu 1337 photodiodes at six Ar^+^ ion laser lines, and he has also measured the oxide-bias correction for a single Hamamatsu 1337 photodiode at four Ar^+^ ion laser lines. Zalewski and Hoyt [4] presented their data, which are summarized in bold face type in table 3, as an intercomparison of spectral responsivity. Since the internal quantum deficiency is the quantity of interest in this paper, it is necessary to compute this quantity from the data reported by Zalewski and Hoyt as shown in table 3. The corrections for nonlinearity that Zalewski and Hoyt derived from reverse-bias measurements also eliminate any effects of recombination in the rear region of the photodiodes.

Fox’s [17] oxide-bias data, which are summarized in table 4, were measured at 10 V, and were reported as a fractional increase in photocurrent *I* given by γ_0_=*I*(10V)/*I*(0) − 1. The oxide-bias experiment reported in Part II shows that the internal quantum efficiency at zero bias δ*_x_* is given by

$$\delta_{x}=1-1/\left(1+1.13\;\gamma_{o}\right),$$ (19)

where the maximum oxide bias-voltage is 10 V. The average internal quantum efficiencies reported by Fox are also shown in table 4, and the internal quantum deficiencies calculated from them are also shown there.

Equation (17) was normalized to the results of Zalewski and Hoyt at λ_f_=441.6 nm, and the predictions of that equation are compared with the results of Zalewski and Hoyt at 633 nm in table 5. Note that δ*_x_*(λ_r_) = 0 for this data set due to the application of reverse bias. Equation (17) was also normalized to the oxide-bias results of Fox at λ_f_=468.18 nm, and the predictions of that equation are compared with the oxide-bias results in table 6. Again note that δ*_x_*(λ_r_) = 0 because oxide-bias measurements are not sensitive to recombination in the rear of the photodiode.

The uncertainty estimates for eq (17) in tables 5 and 6 made use of the data in figure 11 even though those data apply to λ_f_=440 nm rather than 441.6 or 468.2 nm. This does not distort the estimated uncertainty significantly because the terms are added in quadrature, and the first term on the right-hand side of eq (18) is larger than the second term for both sets of data. Fox’s data point at 406.85 nm was included in table 6 even though the uncertainties associated with extrapolating δ_f_(λ) to wavelengths shorter than λ_f_ are very unfavorable for high-accuracy applications. The good agreement at this wavelength must be considered fortuitous considering the uncertainty associated with the predicted value. The differences at the other wavelengths never exceed 0.00015, and they fall within the estimated uncertainty for the differences.

**Figure 13:**
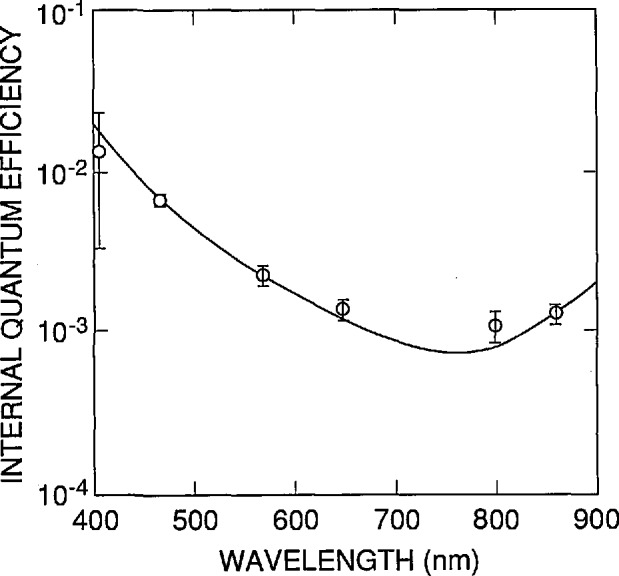
Comparison of measured data of reference [17] (open circles) with eq (17) (solid line) normalized to pass through the measured point at 468.18 and 859.1 nm. At these wavelengths the error bars are the uncertainties in the measured values; at the other wavelengths the error bars are the quadrature sum of the uncertainties in the measured values and those associated with the use of eq (17).

Table 7 and figure 13 compare the internal quantum-deficiency results of Fox [17] with the predictions of eq (17) when normalized at λ_f_=468.18 nm and at λ_r_ = 859.07 nm. The data in figures 11 and 12 that apply for λ_f_=440 nm and for λ_r_ = 860 nm, respectively, are used with eq (18) to estimate the uncertainties assigned to the predictions of eq (17). The difference at 406.85 nm is much larger than that obtained with the oxide-bias data but still falls within the estimated uncertainty. Because the oxide-bias experiment is not sensitive to the quantum yield for electron-hole pair production, it is tempting to imagine that the discrepancy between the oxide bias and internal quantum efficiency reflects the fact that the quantum yield for the 1337 type photodiode is greater than unity at 406 nm. However, the fact that this discrepancy is not statistically significant at the one-sigma level shows that this conclusion cannot be drawn from the data presented here. The differences at the other wavelengths never exceed 0.0003, and only fall outside the estimated uncertainties for the differences at 799.54 nm.

The results shown in tables 5–7 and figure 13 verify that the internal quantum-deficiency spectrum of multiple-reflection radiometers based on Hamamatsu 1337 photodiodes can be interpolated over the 440 to 860 nm spectral region from only two measured quantum deficiencies, one at each end of the region. This greatly reduces the number of measurements needed for high-accuracy spectral calibrations of these photodiodes and radiometers. Furthermore, the theoretical uncertainty analysis suggests that the uncertainties in the interpolated values will be less than or equal to the uncertainties in the measured values, at least as long as the latter are greater than ±0.0001. The results in tables 5 to 7 and figure 13 do not contradict this idea, but the experimental data are not accurate enough to confirm it. On the other hand, the experimental data do confirm that an uncertainty of ±0.0003 is obtainable.

## 4. Conclusion

Part III of this series of papers has shown that Version 2 of the semiconductor-device modeling program PC-1D can be used to model the spectral shape of the internal quantum deficiency of 1337 type photodiodes with very small uncertainty over the 440 nm spectral region. The largest uncertainties are caused by uncertainties in the absorption-coefficient spectrum of silicon, diode-to-diode variations in the shape of the front-region dopant distribution, and diode-to-diode variations in the position-independent rear-region minority-carrier lifetime. It was not possible to estimate the uncertainty caused by approximating the rear-region minority-carrier lifetime as being independent of position.

Simple formulae were derived that allow high-accuracy internal quantum-efficiency calibrations on 1337 type photodiodes to be interpolated from measured values at the ends of the 440 to 860 nm spectral region over the interior of that region. The uncertainties that can be obtained with these formulae are comparable to those that can be obtained with the highest accuracy measurements currently available.

## Figures and Tables

**Table 1:** Recombination related parameters used with the doping distributions shown in figure 3 to model the quantum-deficiency spectra of Hamamatsu 1337 type photodiodes

Case	*N*_ss_ (cm^−3^)	*S* (10^5^ cm/s)	τ_r_ (s)	*N*_A_(*x*)
1	0	0.71264	∞	dashed curve
2	−3×l0^12^	1.83500	∞	dashed curve
3	−1×10^13^	100.000	∞	dashed curve
4	−4×l0^12^	1.56000	∞	solid curve

**Table 2:** Values of the parameters to be used in eq (4) for extrapolating a short wavelength internal quantum-deficiency value to longer wavelengths and in eq (5) for extrapolating a long wavelength internal quantum-deficiency value to shorter wavelengths

Parameter	eq (4)	eq (5)
*A*_0_	1.38002×l0^2^	2.02157×10^−8^
*A*_1_	1.47530×10^2^	3.51764×10^3^
*A*_2_	8.07476×10^−5^	0.0
λ_9_	4.39312×10^1^ nm	7.53991×10^1^ nm
λ_2_	4.27998×10^2^ nm	1.66889×10^8^ nm

**Table 3:** Average values of, and uncertainty estimates for, the internal quantum efficiency ϵ_r_, spectral responsivity *R*, one minus the reflectance (1 – ρ) and nonlinearity eorreetion (1 – NL) reported in reference [4] (bold face type) at wavelength λ for five multiple-reflection radiometers based on 1337 type photodiodes, and internal quantum-deficiency values δ*_x_*(λ) calculated from the reported data

λ (nm)	ϵ_f_	*R* (A/W)	(1 – ρ)	(1 – NL)	δ*_x_*(λ)
441.6	+ **0.99347**				+ 0.00653
	**±0.00040**				±0.00040
633.0	**+0.50840**	**+0.99704**	**+0.99972**	**+0.00097**	
		**±0.00009**	**±0.00014**	**±0.00004**	±0.00023

**Table 4:** Oxide-bias data γ_0_ reported in reference [17] for a single 1337 type photodiode, the average internal quantum-effieiency data *ϵ_x_* reported in reference [17] for ten multiple-reflee-tion radiometers based on 1337 type photodiodes, and the quantum defieieneies δ*_x_*(λ) calculated from these sets of data

λ	oxide bias		iqe	
(nm)	γ_0_	δ*_x_*(λ)	ϵ*_x_*	δ*_x_*(λ)
406.85	+ 0.0126	+ 0.01404	+ 0.9867	+ 0.0133
	± 0.00015	± 0.00017	± 0.0002	± 0.0002
468.18	+ 0.0044	+ 0.00495	+ 0.9934	+ 0.0066
	± 0.00015	± 0.00017	± 0.0003	± 0.0003
568.35			+ 0.9977	+ 0.0023
			± 0.00020	± 0.0002
647.30	+ 0.0007	+ 0.00079	+ 0.9986	+ 0.0014
	± 0.00015	± 0.00017	± 0.0002	± 0.0002
799.54			+ 0.9989	+ 0.0011
			± 0.0002	± 0.0002
859.07	+ 0.0000	+ 0.00000	+ 0.9987	+ 0.0013
	± 0.00015	± 0.00017	± 0.0002	± 0.0002

**Table 5:** Comparison of predictions of eq (17) with measured internal quantum-deficiency data δ*_x_*(λ) in table 3 for λ_f_=441.6 nm and δ_r_(λ_r_) = 0

Prediction wavelength (nm)	Measured value reference [4]	Predicted value eq (17)	Difference
633.0	+ 0.00097	+ 0.00089	− 0.00008
	± 0.00023	± 0.00007	± 0.00024

**Table 6:** Comparison of predictions of eq (17) with internal quantum-defieiency data δ*_x_*(λ) derived from oxide-bias data in table 4 for λ_f_=468.18 nm and δ*_x_*(λ_r_) = 0

Prediction wavelength (nm)	Measured value reference [15]	Predicted value eq (17)	Difference
406.85	+ 0.01404	+ 0.01321	− 0.00083
	± 0.00017	± 0.01144	± 0.01144
647.30	+ 0.00079	+ 0.00090	+ 0.00010
	± 0.00017	± 0.00030	± 0.00034
859.07	+ 0.00000	+ 0.00015	+ 0.00015
	± 0.00017	± 0.00005	± 0.00018

**Table 7:** Comparison of predictions of eq (17) with measured internal quantum-deficiency data in table 4 for λ_f_=468.18 nm and λ_r_ = 859.07 nm

Prediction wavelength (nm)	Measured value reference [15]	Predicted value eq (17)	Difference
406.85 nm	+ 0.0133	+ 0.01744	+ 0.00414
	± 0.00025	± 0.01000	± 0.01000
568.35 nm	+ 0.0023	+ 0.00224	− 0.00006
	± 0.00030	± 0.00007	± 0.00031
647.30 nm	+ 0.0014	+ 0.00121	− 0.00019
	± 0.00020	± 0.00004	± 0.00020
799.54 nm	+ 0.0011	+ 0.00082	− 0.00028
	± 0.00021	± 0.00009	± 0.00023

## Footnotes

1 Certain commercial equipment, instruments, or materials are identified in this paper to specify adequately the experimental procedure. Such identification does not imply recommendation or endorsement by the National Institute of Standards and Technology, nor does it imply that the materials or equipment identified are necessarily the best available for the purpose.
